# Effects of CO_2_ and Temperature on Tritrophic Interactions

**DOI:** 10.1371/journal.pone.0062528

**Published:** 2013-04-25

**Authors:** Lee A. Dyer, Lora A. Richards, Stephanie A. Short, Craig D. Dodson

**Affiliations:** 1 Department of Biology, University of Nevada Reno, Reno, Nevada, United States of America; 2 Lewis & Clark Law School, Portland, Oregon, United States of America; 3 Desert Research Institute, University of Nevada Reno, Reno, Nevada, United States of America; University of California, Berkeley, United States of America

## Abstract

There has been a significant increase in studies of how global change parameters affect interacting species or entire communities, yet the combined or interactive effects of increased atmospheric CO_2_ and associated increases in global mean temperatures on chemically mediated trophic interactions are mostly unknown. Thus, predictions of climate-induced changes on plant-insect interactions are still based primarily on studies of individual species, individual global change parameters, pairwise interactions, or parameters that summarize communities. A clear understanding of community response to global change will only emerge from studies that examine effects of multiple variables on biotic interactions. We examined the effects of increased CO_2_ and temperature on simple laboratory communities of interacting alfalfa, chemical defense, armyworm caterpillars, and parasitoid wasps. Higher temperatures and CO_2_ caused decreased plant quality, decreased caterpillar development times, developmental asynchrony between caterpillars and wasps, and complete wasp mortality. The effects measured here, along with other effects of global change on natural enemies suggest that biological control and other top-down effects of insect predators will decline over the coming decades.

## Introduction

Global change is currently one of the most important issues for ecology and for ecological applications, such as conservation and agriculture [1], [2]. Basic and applied ecologists have made considerable progress towards understanding the effects of rapid changes in biogeochemical cycles, climate, and species distributions [3]–[5]. While most theoretical and empirical predictions of the consequences of such changes are still focused on individual species distributions, an increasing number of studies have tackled the more difficult goal of predicting the effects of these large scale changes on interacting species [4], [6]–[22]. Similarly, the majority of ecological climate change studies examine effects of single climate change variables on biological systems, but some studies have examined interacting effects of different global change parameters, such as increased CO_2_ and increased temperature, on species interactions or on entire biotic communities [23]–[30]. Nevertheless, the theoretical framework for studying climate change and biotic interactions is still underdeveloped [4], [31]–[33] and there are insufficient numbers of empirical studies to effectively guide theory or synthesis [20], [28]–[30]. Existing studies have revealed that the effects of interacting global change parameters on trophic interactions are variable, especially for upper trophic levels, and more studies are necessary to determine factors that alter responses of biotic interactions to climate change [29]. To this end, we examined the effects of changes in temperature and CO_2_ on direct and indirect tritrophic interactions between plants (alfalfa, *Medicago sativa* L., Fabaceae), plant chemistry (sapogenins and saponins), herbivores (caterpillars, *Spodoptera exigua* Hübner, Noctuidae), and natural enemies (parasitic wasps, *Cotesia marginiventris* Cresson, Braconidae).

**Figure 1:**
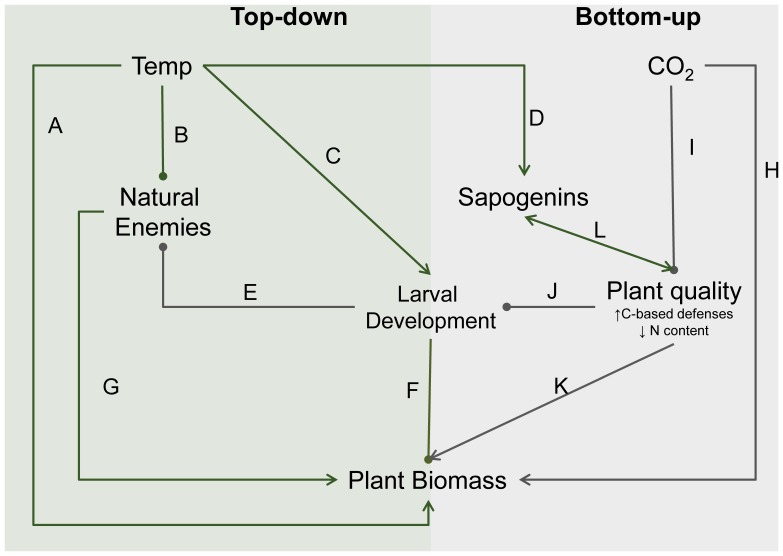
Path diagram summarizing direct and indirect effects of increased temperature and CO_2_ on tritrophic interactions. The letters included as path coefficients correspond to direct effects that have been quantified in previous studies (cited in Table 1). Positive effects between variables are indicated by an arrow and negative effects are indicated by a filled circle. Temperature effects on top-down dominated interactions are in green and CO_2_ effects on chemically mediated interactions are in grey.

Increases in temperature and atmospheric CO_2_ interrupt the relationships between plants, herbivores, and their associated enemies through complex mechanisms [10], [12], [19], [25], [29], [34], but the existing body of literature allows for formulating hypotheses about the combined effects of temperature and CO_2_ on tritrophic interactions (Fig. 1, Table 1). For elevated temperature, tritrophic studies have demonstrated predominately top-down effects on plant biomass by directly altering herbivore and natural enemy development and survival. High temperatures (and extreme weather events) can have direct negative effects on survival of natural enemies, such as parasitic wasps [7], [10], [35]–[37], which are particularly sensitive to any temperature changes [21], [22]. One such negative effect of increased temperatures and heat waves is an increase in parasitoid development times [10], [28]. Elevated temperatures can also decrease larval development time [14], [30], [38], [39] and increase performance [30]. These combined temperature driven changes could negatively affect natural enemies by causing asynchrony between herbivore hosts and parasitoid development, resulting in a greater potential for herbivore outbreaks and lower plant biomass [40].

**Table 1:** Summary of selected studies that have demonstrated direct and indirect effects of increased temperature and CO_2_ on chemically mediated tritrophic interactions, as illustrated in Figure 1. “Path” refers to the letters included as path coefficients in Figure 1.

*Path*	*Relevant direct and indirect trophic interactions*
A	Direct effects of increased temperature on plant biomass are often positive [65].
B	High temperatures can have direct negative effects on natural enemy survival [8]–[9].
C	Elevated temperatures can decrease larval development time [12], [15].
D	Increased temperature can cause increased production of sapogenins [66].
E	Faster developing larvae reduce their window of vulnerability to predators and parasitoids [67].Developmental asynchrony between host and parasitoid can result in high parasitoid larval mortality [68], [69].Changes in larval performance can affect natural enemies; poor host quality negatively affects parasitoids [70].
F	Herbivore larvae consume plant tissues, reducing biomass [71]. This reduction in biomass is increased by other factors: elevated CO_2_ can increase consumption rate and total plant biomass removed, and herbivores can increase consumption rates to compensate for poor leaf quality [19], [20], [24].
G	The trophic cascade. Enemies have an indirect positive effect on plant biomass via controlling heribivory [72].
H	Elevated CO_2_ directly increases plant growth by increasing photosynthesis [15]–[18], [24].
I	Elevated CO_2_ reduces plant quality by increasing some carbon based defenses or by decreasing plant N content [19]–[24].
J	Poor leaf quality due to elevated CO_2_ can negatively affect herbivore performance [20], [26].
K	There is a trade-off between investing resources in plant defense versus growth [34].
L	Saponins are derived from sapogenins and concentrations are positively correlated.

In contrast to the consumer-driven effects of higher temperatures on plant biomass, elevated atmospheric CO_2_ is hypothesized to directly affect plant quality and biomass (Fig. 1). Elevated CO_2_ increases plant growth by increasing photosynthesis [41]–[45] and indirectly by reducing plant quality for herbivores by increasing concentrations of plant secondary metabolites [46]–[48] and decreasing leaf nitrogen content [45], [46], [48]–[50]. While these changes in plant quality can have negative bottom-up effects on herbivore performance [46], [51], [52], they can cause compensatory increases in consumption rates and total feeding by herbivore larvae [45], [46], [50]. Changes in herbivore performance mediated by plant quality can also negatively affect parasitoid performance [53]–[55]. Both the decrease in parasitoid performance and compensatory herbivore feeding cause a decrease in plant biomass, despite the direct increases in plant growth due to elevated CO_2_ (Fig. 1)_._

The studies that have examined effects of climate change on tritrophic interactions [10], [12], [19], [25], [28], [29], [34], [56] suggest that effects on these interactions will favor outbreaks in natural and managed ecosystems, including most agriculture. Most notably, increases in climatic variability and extreme weather events associated with climate change disrupt regulation of herbivores by predators and parasitoids [7], [10], [57], [58]. Based on this literature and any summary of pairwise interactions or direct effects (Fig. 1), it is not possible to make adequate predictions about effects of one climatic variable on trophic interactions because of confounding effects caused by changes in other variables [29], [30]. Including manipulations of multiple global change parameters, in this case, both CO_2_ and temperature, is important in plant-herbivore-parasitoid systems because parasitoid communities are particularly sensitive to temperature changes [21], [22] and this sensitivity could alter responses to increased CO_2_.

It is clear that natural enemies and plant chemistry both can negatively affect herbivore populations in terrestrial systems. But how will these interactions respond to changes of multiple climatic variables [29], [30]? Will the abundance of pest herbivores simply increase due to higher temperatures [14], [30], [38], [39]? Will increases in extreme weather events lead to disruption of normal pest control by natural enemies [7], [10], [57], [58]? Will carbon-based plant defenses increase in response to warming and enhanced CO_2_ [30]? Or will unpredictable patterns emerge due to interactions, synergies, or complex indirect pathways [6], [29], [30], [32]? We addressed these general questions using a model study organism, alfalfa, *Medicago sativa*, which is a high quality forage crop grown worldwide. It is particularly important in the western United States, where it is ranked third in forage crop acreage and accounts for nearly forty percent of the domestic alfalfa production [59]. The saponins and sapogenins found in alfalfa are antiherbivore compounds and can have significant effects on associated arthropods; concentrations of these compounds are affected by a variety of perturbations [60]. The arthropod communities associated with alfalfa are complex, with hundreds of species, diverse trophic assemblages and the potential for dampening or enhancing interactions [60]–[62]. For this study, we examined a simple but biologically significant slice of this food web and addressed specific hypotheses about how changes in temperature and CO_2_ interact to affect relationships between plant chemistry and one species of plant, caterpillar, and parasitoid.

## Methods

### Experimental Overview

The research described here was conducted from January 2006 to December 2009 at Tulane University (New Orleans, LA, USA) and the University of Nevada (Reno, NV, USA). We examined the effects of increased CO_2_ and temperature on alfalfa chemical defenses and biomass, growth and survivorship of the generalist herbivore *Spodoptera exigua*, and growth and survivorship of the parasitoid *Cotesia marginiventris* in chamber experiments. We utilized a full factorial design manipulating temperature (ambient and elevated), CO_2_ (ambient and elevated) and food chain length (plants only, plants+herbivores, plants+herbivores+parasitoids). Thus, alfalfa plants, grown from seeds, were subjected to 12 different treatment combinations. Temperature and CO_2_ were manipulated both independently and simultaneously in sequential experiments with these combinations of treatments: ambient temperature and CO_2_, elevated temperature and ambient CO_2_, elevated CO_2_ and ambient temperature, and elevated CO_2_ and temperature. Ambient CO_2_ was 380 ppm and elevated CO_2_ was 650 ppm, which is within the range of the projected increase in ambient levels by 2080 [63], [64]. Ambient temperatures were the mean monthly high (30°C day) and low (18°C night) temperatures during July (mid growing season) in northern Colorado. The high temperature was 5°C above ambient temperature (35°C day–23°C night), the maximum projected increase due to global warming [63], [64]. The experiments took place inside 3 VWR CO_2_ Incubators, which control CO_2_ and temperature, with the light regime of 14 hour days and 10 hour nights. Levels of CO_2_ and temperature were randomly assigned to each incubator for each trial. Within the incubators, food chain treatments were randomly assigned to separate cages (plastic microcosms, 24×24×32 cm), resulting in interspersion across space and time. There were 30 replicate cages utilized for each treatment combination, for a total of 360 cages. Cages that did not meet conditions of the experiment (i.e. larvae or plants were not ready for introduction of parasitoids or caterpillars) were discarded, and when possible these replicates were repeated at later dates.

### Plants

Each cage contained nine pots with four alfalfa plants each (36 plants total) and when plants were harvested, all plants and associated insects within a cage were combined for biomass and other measures. Pots contained watered soils at the start of experiments, and incubator relative humidity was greater than 80%, so plants were not watered during the course of the experiment. At the end of each experiment, plants were harvested, weighed, and air-dried for analysis of total carbon, total nitrogen, and quantification of secondary metabolites; for these chemical analyses, plants were combined from 10 replicate cages (with the same treatment combinations) to allow for sufficient plant material.

### Insect Colonies

*Spodoptera exigua* were purchased from a supplier (Agripest) and immatures of the parasitoid *Cotesia marginiventris* were collected from northern Colorado fields; both were maintained as colonies in the laboratory. Two newly eclosed (within 12 hours) first instar caterpillars were placed on each alfalfa plant after the appearance of the second trifoliate leaf (approximately 4 weeks after planting). This resulted in 72 caterpillars per cage. In parasitoid addition treatments, the caterpillars were allowed to feed undisturbed for 7 days, at which point a mated female parasitoid was added to each cage and remained in the cages for the duration of the experiment. We ensured that all caterpillars were in the third instar at this point, regardless of experimental treatment. Each trial ended when the last caterpillar pupated or died. Herbivore days to eclose, pupal mass, survival, and parasitism rate were recorded; cage means were calculated for days to eclose and pupal mass, while total survivorship and total percent parasitism for the 72 caterpillars was calculated as a single value for the cage.

### Chemical Analysis

To analyze saponin content, we utilized a modified isolation and quantification procedure [65]. In preparation for chemical extraction, leaf samples were dried overnight in an oven at 40°C and ground to a coarse powder, and subsamples of the dry leaf powder were analyzed by the Nevada Stable Isotopes Lab for total carbon and nitrogen content. One hundred milligrams of dry leaf powder were placed into a centrifuge tube and compounds were extracted from the leaf material in 30 ml of 80% ethanol with stirring. The samples were then centrifuged and the sample plus solvent was separated from the leaf material and dried under a vacuum. The process was repeated to completely extract compounds from the leaves. The dried samples were then dissolved in 15 ml methanol and defatted by shaking the solution with 15 ml of hexanes (98.5% hexane plus a mixture of isomers) in a centrifuge tube. The hexane layer was pipetted off and the process was repeated. The hexanes plus lipids were dried under N_2_ with heat. The defatted methanol layer was dried under a vacuum, and the samples were dissolved in 20 ml water. This solution was centrifuged to separate any remaining leaf material from the dissolved sample.

C-18 SepPak cartridges (Waters Corp.) were then preconditioned with 15 ml acetone followed by 15 ml water. The water with dissolved sample was passed through the cartridge, and the elution was dried under a vacuum. The cartridge was then sequentially eluted with 20 ml each of 35%, 60%, 80% and 100% methanol. The elutions were transferred directly to a pre-weighed scintillation vials and dried under N_2_ with heat. Samples were stored in the freezer. According to previous work with this method, the 35% fraction contains flavans, the 60% fraction is comprised of flavones, the 80% fraction contains saponins and the 100% fraction contains sapogenins. The water fraction has sugars and organic acids, and the hexanes have lipids. Samples were completely dried overnight in an oven at low temperature. Vials with samples were then weighed to determine the mass of each class of compounds contained in the leaf material. The weights of samples from the elutions were used in analyses.

Content of elutions was confirmed by HPLC with a matrix-assisted laser desorption ionization source. The main components for the 80% elution fraction were the alfalfa saponins soyasapogenol B-3-O-Rhamnose-Galactose-Glucose carboxylic acid (mass is 965.5 for the sodiated ion +H) and Hederagenin-3-O- [Beta D glucose acid methyl ester] -28-O- [Beta D glucose] (mass is 847.5 for the sodiated ion +H). The main components for the sapogenin (100%) fractions were hederagenin (mass is 685.5 for the ion and 494.6 for the sodiated ion +H), and zahnic acid (mass is 508.6 for the sodiated ion +H), medicagenic acid (mass is 522.6 for the sodiated ion −2 H+).

### Statistical Analysis

The focal statistical analyses were path analyses based on our causal hypotheses presented in Figure 1. In order to identify which variables were best to test in our focal path analyses, the main and interaction effects of temperature, CO_2_ and parasitoid treatment on all response variables were estimated using analysis of variance (ANOVA). For ANOVAs, replication for plant chemistry was lower than for other response variables because plants from different cages (replicates with the same levels of all treatments) were combined to provide enough material for chemical analysis. Cage means of caterpillar and plant response variables, including survival and percent parasitism (for 72 caterpillars), were used as response variables, and residuals from these variables met assumptions of normality. In addition, we used a Mann-Whitney z statistic to test the specific hypothesis that percentage parasitism was associated with the 4 combinations of temperature and CO_2_.

We examined direct and indirect effects of CO_2_ and temperature on alfalfa biomass with path analysis (Proc CALIS, SAS Institute Inc., NC). We proposed 2 general models based on previous literature (Fig. 1) as well as an alternative, simpler model, all of which elucidated direct versus indirect effects of CO_2_ and temperature on alfalfa biomass, quality, larval development, and parasitoid performance. While other important interaction pathways, including numerous indirect effects, can be proposed from the literature, several reviews suggest that the direction and magnitude of those effects are still too variable to predict [20], [29], [30]. Path models yielding a goodness of fit chi-square with a *P*-value greater than 0.5 were considered a good fit to the data.

## Results

**Figure 2:**
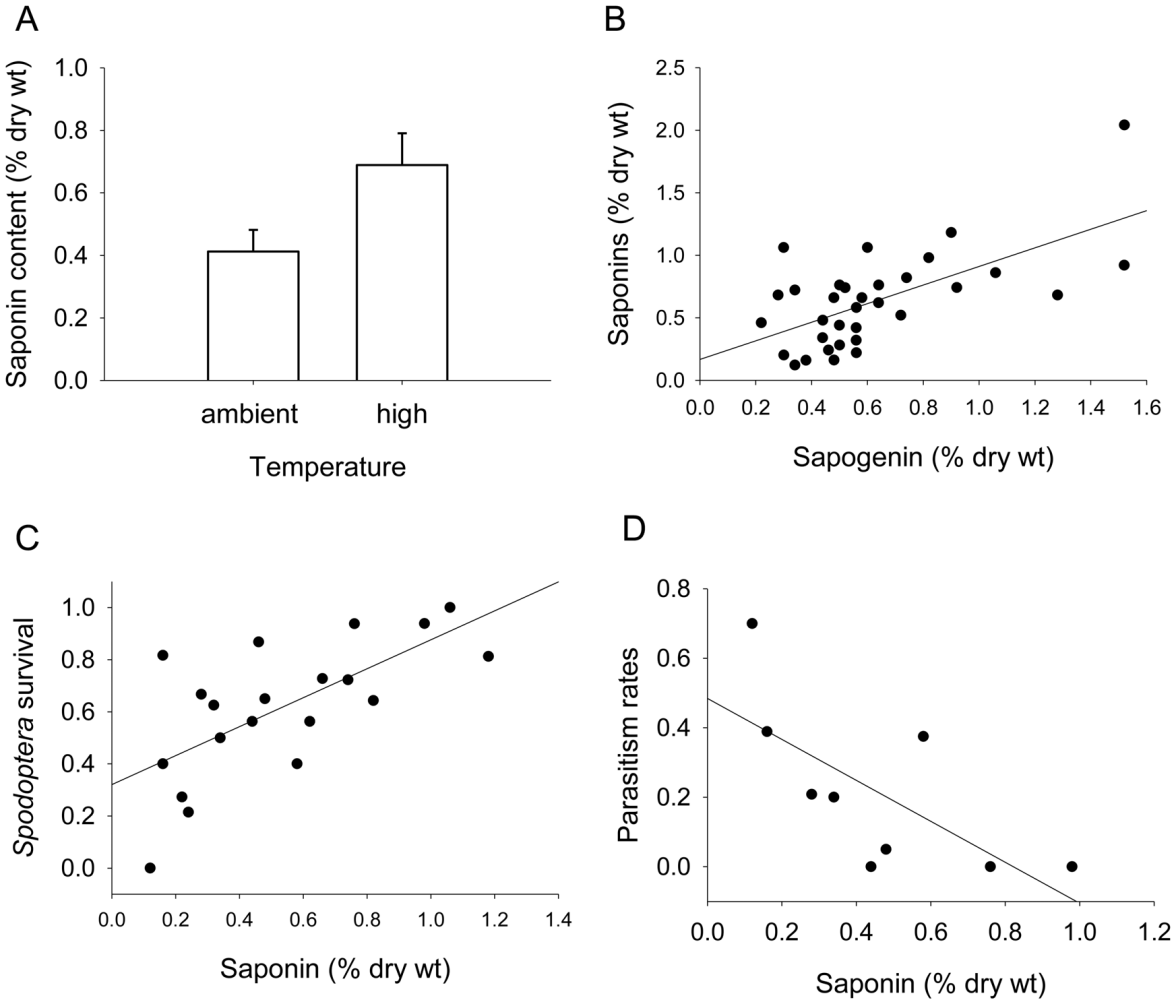
Saponin content in alfalfa (A, B) and the effects on *Spodoptera* survival (C)(R^2^ = 0.379 F_1,19_ = 10.964 P<0.01) and parasitism rates (D)(R^2^ = 0.477 F_1,8_ = 6.383 P<0.05). Values in panel A are mean saponin content (±1 SE) under ambient and high temperatures; ANOVA, F_1,22_ = 4.762, P<0.05. The sapogenins in panel B are biosynthetically unrelated to the corresponding saponins; R^2^ = 0.40, F_1,33_ = 21.351, P<0.001.

The effects of temperature on plant quality were consistent with predictions of overall decreases in plant quality with projected climate change. Warmer temperatures caused an increase in saponin content (Fig. 2), which was positively correlated with the concentration of metabolically unrelated (i.e. these compounds were not simply the same saponins without the sugars) sapogenins (Fig. 2). In contrast, based on ANOVA, there were no significant effects of CO_2_ on saponins or sapogenins and there were no complex interactions. Also based on ANOVAs, there were no significant effects of temperature and CO_2_ on carbon and nitrogen content, C: N, lipids, flavones, sugars, and flavans. Saponin content was correlated with both herbivore and parasitoid success: *Spodoptera* survival was positively associated with saponin content, while parasitism rates were negatively associated with saponin content (Fig. 2).

**Figure 3:**
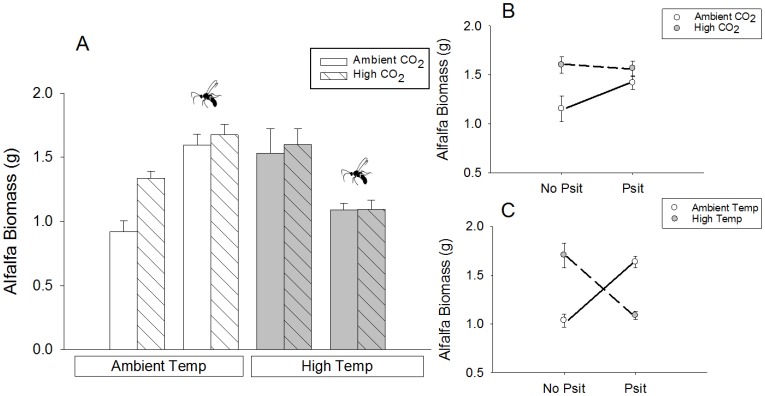
Alfalfa biomass with herbivores under ambient and elevated temperatures and CO_2_. (**A**) Mean alfalfa biomass (±1 SE) under different treatments; the wasp icon indicates the parasitoid addition treatments. There was a significant effect of CO_2_ on alfalfa biomass; F_1,195_ = 10.30, P<0.01. (**B**) The interaction between CO_2_ and parasitoid treatment (Psit); F_1,195_ = 8.02, P<0.01. (**C**) The interaction between temperature and parasitoid treatment; F_1,195_ = 51.57, P<0.001.

**Figure 4:**
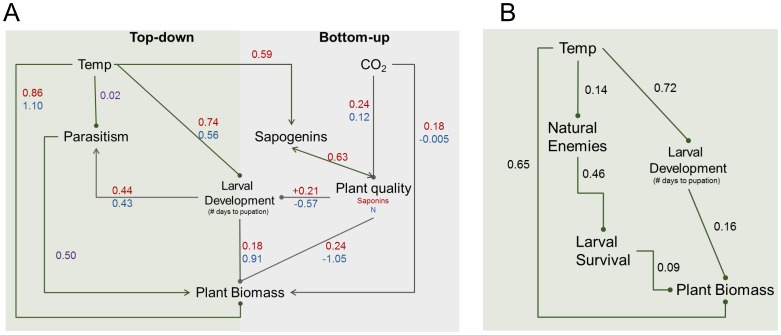
Path diagrams summarizing 2 models of direct and indirect effects of temperature and CO_2_ on alfalfa biomass. Positive effects between variables are indicated by an arrow and negative effects are indicated by a filled circle. Standardized path coefficients are from the two general path models – blue coefficients are from a model that examined total leaf nitrogen (N) as a plant quality variable and red coefficients are from a model that examined saponins and sapogenins as plant quality variables. (**A**) Temperature and CO_2_ effects on plant chemistry and the effects on alfalfa biomass. (**B**) A nested path model that demonstrates that the positive effects of natural enemies on plant biomass are mediated by decreased larval survivorship.

Complex interactions between abiotic and biotic factors were most evident when examining alfalfa biomass as the ANOVA response variable. The indirect positive effect of parasitoids on alfalfa biomass (i.e. the trophic cascade) had the strongest effect on alfalfa for all combinations of temperature, CO_2_, and parasitoid presence (Fig. 3); the lowest mean biomass per microcosm was found under conditions of no parasitoids+ambient temperature+ambient CO_2_. However, the positive effects of parasitoids on alfalfa biomass were attenuated with increasing CO_2_ (Fig. 3B) and disappeared with increasing temperature (Fig. 3C). These significant interactions were corroborated and refined by our path analyses (Fig. 4), which generally supported predictions from previous literature (Fig. 1).

The path analyses, which were focused on effects on biomass and saponin/sapogenins, helped clarify potential mechanisms for the strong negative effect of temperature on alfalfa biomass. Both general models included a pathway that incorporates the known tradeoff between plant quality (saponin production or total N content) and growth (Pearson *et al.* 2008)(Saponin, goodness of fit, χ^2^ = 4.66, *df = *8, P = 0.79; N content, goodness of fit, χ^2^ = 2.52, *df = *4, P = 0.64, Fig. 4A). The strong indirect negative effects of temperature on alfalfa biomass via increased saponin content (path coefficients in Fig. 4A) only partly explained the negative effect of temperature on biomass, since there was still a strong direct effect of temperature. The direct and indirect negative effects of temperature on plant biomass were also attenuated by the increase in larval development at higher temperatures and the indirect effects of parasitism (via larval development). The apparent trophic cascade (“direct” effect of parasitism in 4A) was confirmed by the path analysis focused on effects of parasitism on larval development (goodness of fit, χ^2^ = 0.01, *df = *1, P = 0.80, Fig. 4B).

**Figure 5:**
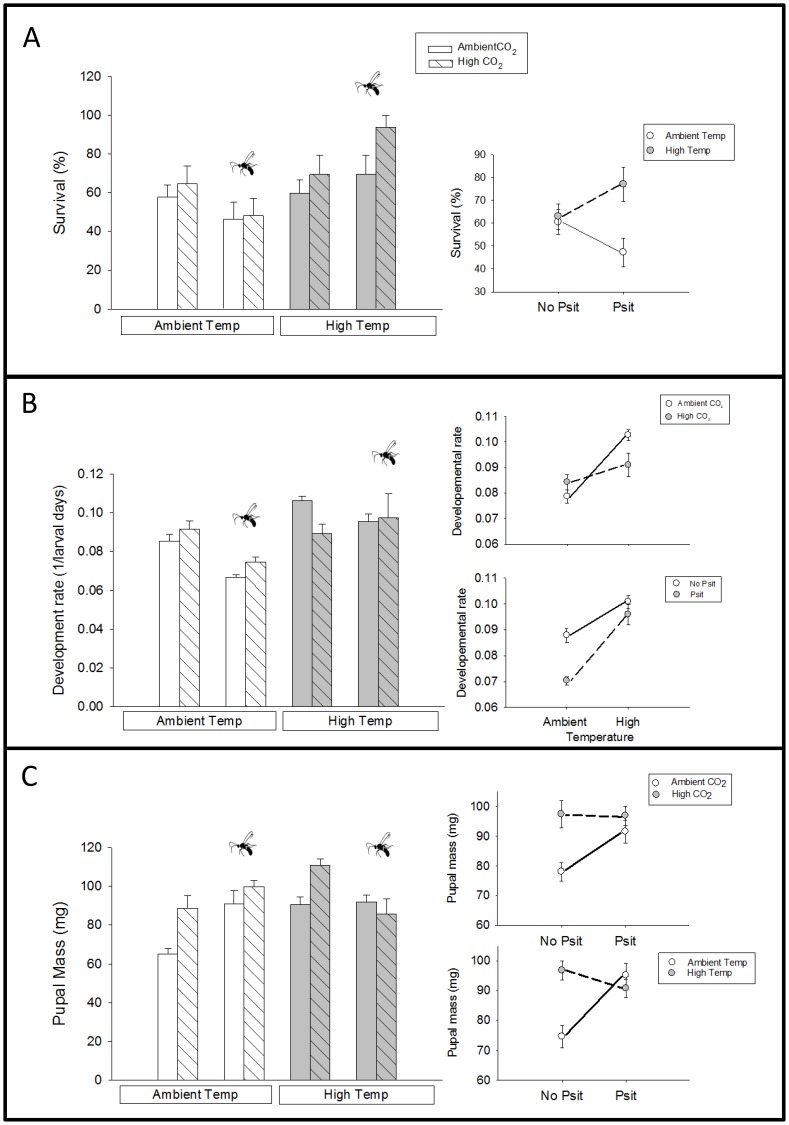
Differences in *Spodoptera* performance between treatments; significant interactions are graphically summarized in the smaller figures to the right. (**A**) Significant temperature (F_1,145_ = 32.63, P<0.001) and parasitoid addition (F_1,145_ = 9.56, P<0.01) effects on larval developmental rates (mean number of days until pupation^−1^± SE) and interactions between temperature and parasitoid addition (F_1,145_ = 7.41, P<0.01) and temperature and CO_2_ (F_1,145_ = 5.58, P<0.05). (**B**) Significant temperature (F_1,196_ = 4.48, P<0.05) and CO_2_ effects (F_1,196_ = 7.85, P<0.01) on pupal mass (mean ± SE) and interactions between temperature and parasitoid presence (F_1,196_ = 13.51, P<0.001) and CO_2_ and parasitoid presence (F_1,196_ = 6.22, P<0.05). (**C**) Significant temperature effect (F_1,195_ = 7.83, P<0.01) on survival (mean ±1 SE) and interaction between temperature and parasitoid presence (F_1,195_ = 5.187, P<0.05).

*Spodoptera* survival, development, pupal mass and parasitism rates were significantly affected by temperature. In the presence of parasitoids, larval survival was the highest under high temperatures and the lowest under ambient temperatures (Fig. 5). All larvae developed faster with increased temperatures, however this increase was dampened by elevated CO_2_ (Fig. 5). Under ambient temperatures, larvae developed significantly slower in the parasitoid treatments – these development results only included *Spodoptera* larvae that pupated, so development rates of individuals that died from parasitoids could not be included in these calculations. The negative effect of parasitoids on herbivore developmental rates disappeared at elevated temperature (Fig. 5).

Both temperature and CO_2_ significantly increased *Spodoptera* pupal mass (Fig. 5) and there were significant interactions with parasitoid presence. Under both ambient temperature and CO_2_, larvae from the parasitoid treatments that survived to pupation had significantly greater pupal masses than those without parasitoids. However, elevated temperature and CO_2_ diminished this effect of parasitoid presence on the pupal mass of surviving individuals.

High temperature and CO_2_ caused dramatic reductions in parasitism (Mann-Whitney, Z = −2.949, P = 0.003), with levels declining from 29.6±8.1% at ambient temperature and CO_2_ to zero at elevated levels of both variables. Even at low CO_2_, increased temperature had strong negative effects on parasitism, causing decreases from 26.9±8.3% to 2.8±2.8% parasitism at high versus low temperature.

## Discussion

The most notable result of our experiments was the indirect effect of increased temperature, which caused decreased development times for caterpillars, resulting in a dramatic negative effect on parasitism. Caterpillars developed rapidly at the higher temperatures and pupated before parasitoids were able to eclose from the late larval stages, resulting in death of the developing parasitoid. This developmental asynchrony could have been exacerbated by the timing of parasitoid introduction to the chambers – had they been introduced earlier, perhaps parasitoid success would have been higher in all treatments. However, the time of introduction was chosen to maximize parasitism based on the phenology of the lab colony – introduction of adults at third instar was optimal for successful parasitism for this particular colony. The 29.6% decline in parasitism recorded at the higher temperature is biologically significant [58] and such developmental mismatches can contribute to overall phenological asynchrony, since the parasitoid must pupate before its host, and if it does manage to eclose, this species has a very short adult stage for mating and finding an early instar host.

In contrast to temperature, increases in CO_2_ indirectly increased larval development times by decreasing plant quality, both of which were associated with lower levels of parasitism. For both temperature and CO_2_ effects on trophic interactions, the host-parasitoid developmental mismatch could contribute to the phenological asynchrony predicted by other climate change scenarios [40] and is likely to synergize with similar global change parameters that delink parasitoids from their hosts. For example, extreme weather events, such as floods and droughts, are increasing with global warming, and these climatic events are likely to cause decreases in caterpillar parasitism rates due to delinking the phenologies of host-parasitoid populations [58]. The fact that increased temperature and CO_2_ each cause temporal developmental shifts between parasitoids and hosts provides a clear mechanism by which alfalfa biomass is not enhanced by parasitoids under a changing climate: parasitoids simply cannot track the variable development and quality of their hosts.

Interestingly, rather than increasing alfalfa biomass indirectly via killing their caterpillar hosts, parasitoids at high temperature caused a decrease in biomass via increased consumption by their caterpillar hosts with no associated mortality. In any biotic community, this effect on biomass could be maintained by immigration of mated adults from adjacent patches or by host shifts by other parasitoids, but in the absence of genetic variation in development rates, the parasitoids would go locally extinct and the direct effects of herbivory on plant biomass would be more important. This parasitoid-biomass result is more relevant to classical biological control, since the continual release of parasitoids could sustain this indirect negative effect on biomass in warmer and CO_2_ enriched conditions. Literature syntheses on climate change and biological control [5], [27] indicate that parasitoid-host developmental mismatches could be common.

Herbivore populations are affected by a combination of abiotic factors, natural enemies, and plant quality and availability. Changes in climate have direct effects on the autecology of herbivores via changes in growth rate, metabolic activity, survivorship, and related factors, but as shown in our chamber experiments, indirect effects can modify significantly the outcomes of consumer-resource interactions [66]. At first glance, the predictions from previous studies on associations between climate variables and consumer-resource relationships are adequate for predicting more complex interactions (*i.e.* comparing Figs. 1 and 4). However, quantifications of only the direct effects do not uncover important indirect mechanisms, such as developmental asynchrony and trade-offs between growth and plant quality. Experiments on simple tritrophic systems that include controlled manipulations of multiple variables that are changing globally provide data that allow for considerable insight into basic questions about the regulation of herbivore populations. More experiments, coupled with observational data and models, will help clarify the conditions under which factors like connectance, parasitism, herbivore, outbreaks, and ecosystem services will increase or decrease in response to interacting climate change variables [8]–[28]. Furthermore, understanding relationships between global climatic changes and tritrophic interactions is particularly important in agricultural systems, where herbivore outbreaks are predicted to increase.

There are multiple consequences to the fact that the responses of biotic interactions to climate change are complex. This includes the possibility that effects acting via different direct or indirect pathways could cancel each other out or lead to changes not predicted by single factor models or experiments. For example, increases in plant biomass due to temperature can be counteracted by changes in parasitism, larval development, and increases in production of secondary metabolites, such that the overall effects of temperature (direct plus indirect) are negative (e.g., Fig. 4). It is clear that such interactions must be examined in order to produce realistic predictions for future impacts of climate change on biotic communities.

Our results are limited to an unnatural experimental setting; most notably, the community is an unrealistically simple chain, the three trophic levels did not evolve together, and levels of temperature and CO_2_ will gradually increase to our experimental levels over a number of decades. However, models and experiments are necessarily artificial, and in this case our experiments provided relevant insight into mechanisms by which trophic asynchrony can occur. Based on our experimental results here and accompanying models [4] and observational field studies [5], [30], [58], we conclude that any efforts to conserve natural enemies or to enhance natural biological control will be negatively affected by complex interactions between multiple climate change metrics and biotic communities. These effects are likely to be exacerbated by increases in extreme weather events [7], [10], [11], [58], contributing to increased insect outbreaks through a number of direct and indirect pathways.
